# A validated LC–MS/MS method for simultaneous determination of key glucocorticoids in animal hair for applications in conservation biology

**DOI:** 10.1038/s41598-023-49940-2

**Published:** 2023-12-28

**Authors:** Ilona Sadok, Kinga Ożga, Daniel Klich, Wanda Olech, Dagny Krauze-Gryz, Agata Beliniak, Rafał Łopucki

**Affiliations:** 1 Department of Chemistry, Institute of Biological Sciences, Faculty of Medicine, The John Paul II Catholic University of Lublin, Konstantynów 1J, 20-708 Lublin, Poland; 2 Department of Biomedicine and Environmental Research, Institute of Biological Sciences, The John Paul II Catholic University of Lublin, Konstantynów 1J, 20-708 Lublin, Poland; 3 Department of Animal Genetics and Conservation, Warsaw University of Life Sciences, Ciszewskiego 8, 02-786 Warsaw, Poland; 4 Department of Forest Zoology and Wildlife Management, Warsaw University of Life Sciences, Nowoursynowska 159, 02-776 Warsaw, Poland

**Keywords:** Biochemistry, Chemical biology, Zoology, Biomarkers, Chemistry

## Abstract

A new method for the determination of main glucocorticoids (cortisol, cortisone, and corticosterone) in hair by liquid chromatography-tandem mass spectrometry was developed. Glucocorticoids were extracted from hair shafts using methanol followed by solid-phase extraction. A validation test was performed using hair from three species of wild mammals with different body size (0.2–800 kg), lifestyle (terrestrial, burrowing and arboreal species), social organization (living in herds or solitary), and different predicted type of hair glucocorticoids: European bison (*Bison bonasus*), European hamster (*Cricetus cricetus*), and Eurasian red squirrel (*Sciurus vulgaris*). Regardless of the species evaluated, the method shows good linearity for all analytes accompanied by satisfactory accuracy (91–114%) and precision (RSD < 13%). Depending on the analyte and hair origin, the calculated limits of quantification were between 0.05 and 1.19 ng/mL, which corresponds to 1.28–31.51 pg/mg. Using cortisol and cortisone as examples, we have demonstrated that measuring multiple glucocorticoids simultaneously provides more comprehensive information than solely concentrating on one, thereby contributing to a more balanced and reliable interpretation of the acquired results. However, the utility of cortisol metabolites as markers of stress response in keratinized tissues should be substantiated by additional experimental studies on targeted animals. We posit that this paper could serve as a crucial catalyst to prompt such experiments.

## Introduction

Glucocorticoids, such as cortisol (11,17,21-trihydroxypregn-4-ene-3,20-dione) and corticosterone (11,21-dihydroxy-4-pregnene-3,20-dione) are well-recognized biomarkers of stress and welfare in human and animals^1–4^. Cortisol is released from the adrenal gland and is regulated via the hypothalamic–pituitary–adrenal (HPA) axis. By the activity of 11-hydroxysteroid dehydrogenases, cortisol is reversibly transformed to its inactive metabolite—cortisone (17,21-dihydroxy-4-pregnene-3,11,20-trione). Corticosterone is the precursor of aldosterone and activates both mineralocorticoid and glucocorticoid receptors. The activation of glucocorticoid metabolic pathways exhibits clear species specificity: corticosterone primarily plays a role in the stress response in reptiles, birds, and rodents, whereas cortisol and cortisone are predominant in other mammals, including humans^1^.

Monitoring of changes in levels of glucocorticoids in blood, saliva, or urine is a well-established strategy for linking psychological and health-related processes in medical, veterinary, and conservation studies^5–7^. Analysis of this kind of sample, however, allows for investigation of a time-point or short-term patterns of steroid hormones secretion and may be influenced by many concomitant factors, including the effect of sampling^8–10^. For this reason, matrices are also sought to determine the long-term, average glucocorticoids level of animals^11^.

Numerous studies have proven that keratinized tissues such as hair or claws can be useful matrices for the determination of glucocorticoids from the last weeks or months^9,12–14^. It is known that glucocorticoids can diffuse from the bloodstream into the hair and then diffuse throughout the shaft but the kinetic of this process depends on the species basis and the thickness of the hair^15^. This variability makes it impossible to establish uniform experimental conditions for assessing the concentration of steroids in keratinized tissues across different animal species, and experimental protocols validated for one species may not yield reliable results for another. It should also be noted that the measurement of glucocorticoids can serve as a basis for implementing specific practical measures to improve the well-being of the studied population. Such circumstances arise, for example, in conservation biology, where the physiological state and welfare of animals form the foundation for implementing specific (often costly) conservation measures^16^. In this context, a reliable quantitative measurement of the long-term concentration of steroids in a given matrix is crucial.

Enzyme-linked immunosorbent assay (EIA) is frequently used to measure endogenous steroid contents in various biological samples due to its rapidness and simplicity^17,18^. However, this technique allows for the monitoring of a single analyte (e.g. cortisol), suffers from poor specificity due to cross-reactivity with other endogenous steroids and lipids and limited dynamic range^8,19–21^. Paradoxically, the cross-reactivity of antibodies in EIA assays can be considered a useful attribute, since it enables the simultaneous detection of glucocorticoid metabolites with a similar chemical structure, which might be present in higher concentrations than the native (unmetabolized) compound within the sample. This approach, focusing on group determination of metabolites rather than native compounds, has found widespread application in studies examining fecal samples from various species of wild animals^22^. It is worth noting that selecting an inappropriate EIA for analysis may result in inaccurate representations of adrenocortical activity, as was for example observed in a study with hair samples^23^.

Besides EIA, numerous LC–MS/MS methods have been developed to measure steroid hormones (including glucocorticoids) in body fluids and hair extracts with good accuracy, precision, and selectivity^12,18,20,21,24–26^. The LC–MS/MS measurement usually requires a small sample volume or amount and allows for the determination of numerous analytes in a single analytical run. However, LC–MS/MS analysis of keratinized tissue samples is known to be challenging due to low endogenous levels of steroid hormones and strong signal suppression caused by co-extracted interfering compounds (so-called matrix effect, ME). Thus, existing LC–MS/MS protocols were devoted to optimizing the extraction of steroid hormones from human^24,25^ or animal hair samples^10^, but there are still significant methodological gaps that make research on different groups of animals difficult. These gaps relate in particular to the possibility of applying the achievements of modern analytics (commonly used, for example, in medicine) in the study of wild and endangered animal species in order to better understand their physiological condition and to make their conservation more effective^27^.

**Figure 1:**
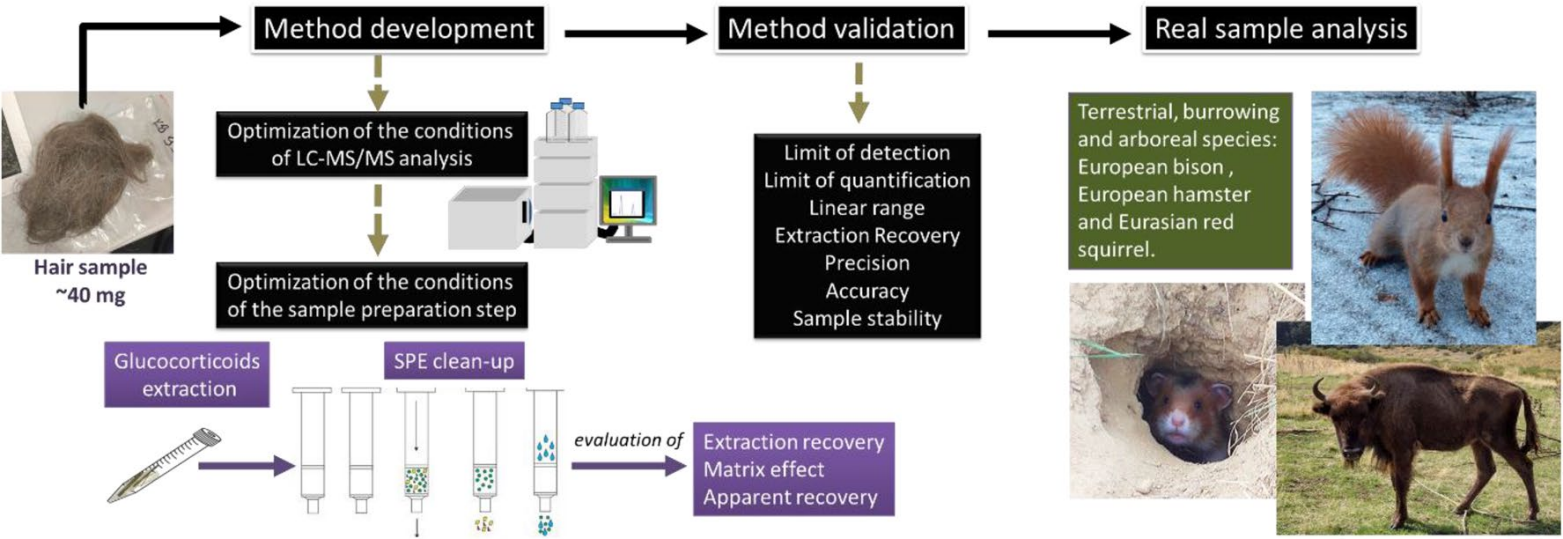
Scheme summarizing the crucial aims of the work.

The overarching goal of this work was to develop and validate the new ultra-high performance liquid chromatography–electrospray ionization–tandem mass spectrometry (UHPLC-ESI–MS/MS) method for simultaneous quantification of cortisol, cortisone, and corticosterone in hair collected from wild animal species (Fig. 1). As a target species, three mammals of different taxa (European bison *Bison bonasus*, Eurasian red squirrel *Sciurus vulgaris*, and European hamster *Cricetus cricetus*), with different body size (0.2–800 kg), lifestyle (terrestrial, burrowing and arboreal species), social organization (living in herds or solitary) and different predicted type of hair glucocorticoids were selected. The selected species are protected in numerous European countries, and extensive research in the fields of ecology and conservation biology is being conducted for them.

We believe that the development of new methodological protocols for determining glucocorticoids in the hair of selected species will allow us to better understand the condition of individual populations and conduct their more effective conservation. Performing such studies on hair samples will allow us to assess long-term glucocorticoid levels and obtain important information about free-living animals with minimal impact on the study organisms. At the same time, we believe that the development of validated LC–MS/MS methods for various groups of wild animals will allow for the dissemination of such analyses in conservation biology.

## Results and discussion

### Optimization of LC–MS/MS parameters

Key settings of tandem mass spectrometer used for ions generation and detection were carefully optimized. The optimal conditions for the simultaneous detection and quantification of glucocorticoids in a multiple reaction monitoring (MRM) mode are presented in Table 1.

**Table 1:** MRM transitions and MS parameters for glucocorticoids and the internal standard.

Analyte	Precursor ion (*m/z*)	Product ion (*m/z*)	FV [V]	CE [eV]	RT [min]	ΔRT [min]
Cortisone	361.1	163	160	24	5.1	2
		121		36		
Cortisol	363	121.2	80	26	5.5	2
		97.5		36		
Cortisol-D_4_*	367.1	121.1	80	26	5.5	2
Corticosterone	347.1	329.1	100	12	6.4	2
		311.1		12		

MS conditions: polarity, ESI + ; nebulizer pressure, 35 psi; drying gas temperature, 300 °C; drying gas flow, 8 L/min; sheath gas temperature, 300 °C; sheath gas flow, 10 L/min; capillary voltage, 4500 V; nozzle voltage, 300 V.

*FV* fragmentor voltage; *CE* collision energy; *RT* retention time.

*internal standard.

**Figure 2:**
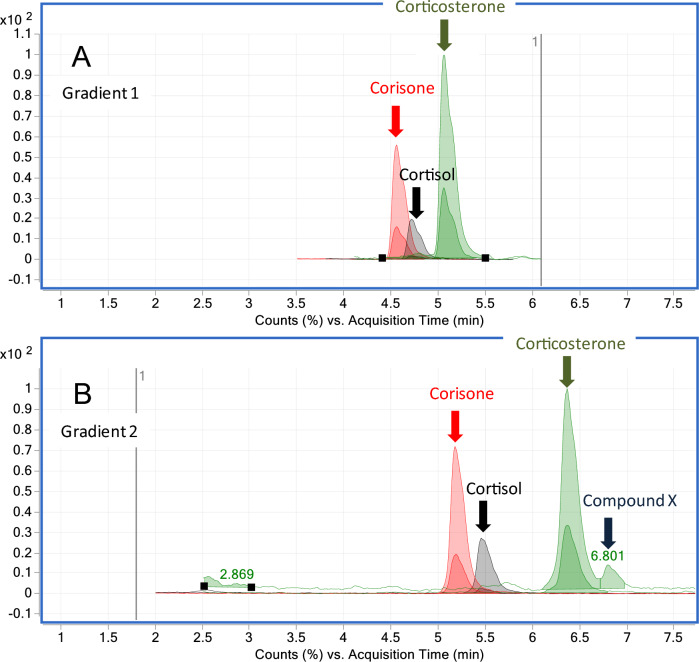
Comparison of LC–MS/MS data acquired during the analysis of the same European bison hair sample fortified with cortisol, cortisone, and corticosterone for different gradients of the mobile phase. Gradient 1: 0–6 min: 10–90% solvent B; 6–9 min: 90% solvent B (post run: 2 min). Gradient 2: 0–4 min: 20–60% solvent B; 4–5 min: 60% solvent B; 5–7 min: 60–70% solvent B (post run: 2 min). Composition of the mobile phase: 0.01% (*v/v*) formic acid in water (solvent A) and methanol (solvent B) (flow rate: 0.35 mL/min). Hair samples were prepared following the optimized sample preparation protocol.

After the optimization of the hair preparation procedure, the gradient of the mobile phase was further adjusted. Analysis of randomly selected European bison hair samples indicated that for this species corticosterone signal is prone to interference caused by unknown compounds. The decrease in the steepness of the mobile phase gradient yields satisfactorily resolved peaks for the target compound and other components of the hair matrix (Fig. 2).

### Development of sample preparation protocol

Next, we adjusted various conditions of the sample preparation protocol to ensure the simultaneous extraction of these three important glucocorticoids in a single extraction step and their reliable quantification in hair samples from different animals. Preliminary studies showed that hair from European bison has the lowest content of studied glucocorticoids compared to other evaluated species (red squirrel, European hamster). Furthermore, it was possible to find a hair-blank sample (containing analytes at an undetectable level) in the amount required for the whole optimization process. The sample preparation step was optimized using hair extracts fortified with cortisol, cortisone, and corticosterone in appropriate concentrations alongside corresponding blank samples.

Prior extraction of glucocorticoids, hair samples from animals were washed twice with isopropanol. This allows for the removal of external contaminants (including cortisol deposited from sweat or sebum) and represents a standard step of hair preparation of many existing protocols^19,28^. However, methanol might be also used for this purpose^23^. To date, a double wash of hair shafts with isopropanol allows for the effective removal of external cortisol with minimal effect on its fraction within the internal hair matrix^19^.

The “gold-standard” method used for the extraction of different glucocorticoids (including cortisol, cortisone, and corticosterone) from the hair is overnight incubation of the sample with methanol^21,25,29,30^. Herein, this strategy was also employed. Conditions of the sample preparation protocol were adjusted for ~ 40 mg of the animal hair sample. Available LC–MS/MS protocols for steroid hormones usually require from 25 mg (horse) or 40 mg (red deer) to 1 g (polar bear) of hair for analysis^21,28,30^.

**Figure 3:**
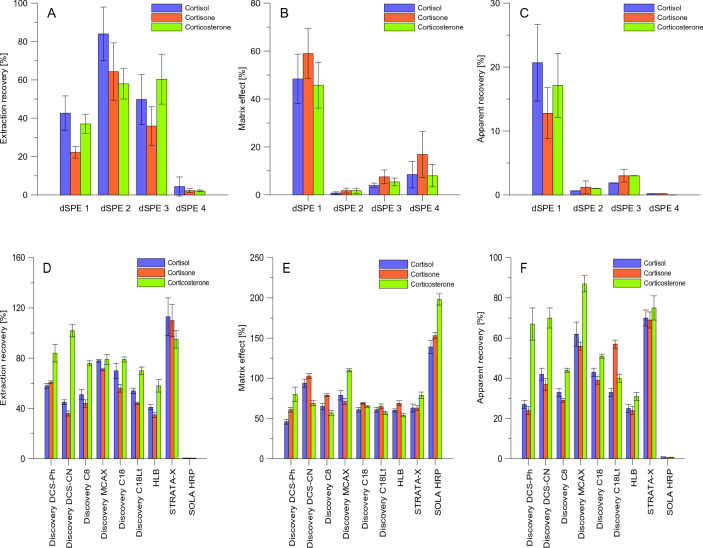
Comparison of different strategies for clean-up of animal hair extracts. Experiments were conducted using 800 µL of blank or fortified with analytes (0.05 µg/mL) methanolic European bison hair extracts. For dSPE (**A**-**C**), an extract was transferred to dSPE tube with a proper sorbent mixture, vortexed for 1 mL, centrifuged (13 000 rpm, 10 min), filtered using a syringe filter (0.22 µm, regenerated cellulose), and analyzed by LC–MS/MS. For SPE (**D**-**F**), a hair extract was evaporated to dryness and reconstituted using 1 mL of water before loading on the cartridge. Applied SPE conditions: conditioning—methanol (1 mL), equilibration—water (1 mL), loading sample (1 mL), washing—water (1 mL), elution—methanol/acetonitrile/3 mol/L acetic acid (40:40:20, *v/v/v*, 1 mL). For SOLA HRP cartridge: washing—5% (*v*/*v*) methanol in water (1 mL), elution—methanol (1 mL). The eluate was evaporated to dryness and redissolved in 200 µL of methanol before analysis.

Two different strategies were evaluated to ensure adequate clean-up of the methanolic extract of animal hair before the determination of glucocorticoids: solid-phase extraction (SPE) and dispersive solid-phase extraction (dSPE). To this, the effectiveness of extract clean-up using four different commercially available dSPE sorbent mixtures and nine SPE cartridges providing different retention mechanisms was evaluated by determining extraction recoveries (R_E_), apparent recoveries (ApR_E_), and matrix effect (ME). Assessment of these parameters gives information about the potential loss of analytes during sample preparation (R_E_), the influence of the co-extracted hair matrix component on the ionization of the analytes in ESI source (ME), and the overall method efficiency (ApR_E_). ApR_E_s were calculated by dividing the glucocorticoid signal in a pre-extracted fortified blank hair sample and that in pure methanol. The obtained data are included in Fig. 2. Generally, noticeably better ApREs and less suppression of analyte ions were reported for SPE than dSPE purification. Regarding R_E_s and ApR_E_s, clean-up of hair extracts using SOLA HRP SPE cartridges and dSPE 4 (sorbent mixture containing MgSO_4_, primary secondary amine (PSA), graphitized carbon black (GCB), and C18) was unsuitable for the determination of glucocorticoids. Sample purification on STRATA-X (containing polymer-based SPE sorbent—a surface-modified styrene divinylebenzene) resulted in the best R_E_s for all studied analytes, and high ApR_E_s compared to other SPE cartridges. The type of SPE cartridges has been so far applied for the LC–MS/MS quantification of urinary cortisol, cortisone, and their tetrahydro-metabolites^20^. In agreement with other research data^20^, STRATA-X cartridges were found to be more adequate for analytes determination than HLB cartridges, which also contain modified styrene polymer as a packed bed. In the case of corticosterone, clean-up using Discovery MCAX, DCS-Ph, DCS-CN cartridges provided better ApR_E_s than Discovery C8, C18, and C18Lt once. Clean-up of hair extract using Discovery MCAX cartridge also resulted in better ApR_E_s for cortisol and cortisone compared to other tested SPE cartridges (except STRATA-X for both analytes, and Discovery C18Lt for cortisone) (Fig. 3).

**Figure 4:**
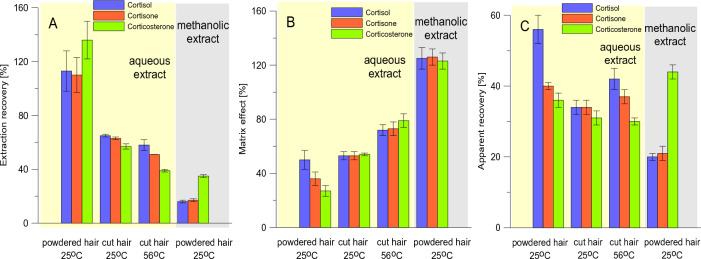
Effect of different strategies of analytes extraction and extract pretreatment on extraction recovery (**A**), matrix effect (**B**), and apparent recovery (**C**). A representative sample of European bison hair was cut into small segments with scissors, and transferred into tubes (40 mg), and directly subjected to extraction (“cut hair”) or further grounded using a standard procedure before extraction (“powdered hair”). Next, analytes were extracted using 1 mL of methanol for 24 h at room temperature (see-saw rocker) or 56 °C (thermoblock). The supernatants (800 µL) were loaded on SPE cartridge (“methanolic extract”) or evaporated to dryness and reconstituted with 1 mL of water (“aqueous extract”) before SPE purification. SPE was performed on STARTA-X cartridges and further treated as specified in Fig. 2. Experiments were carried out using blank samples and fortified with glucocorticoids at 0.05 µg/mL before extraction.

A series of experiments were then performed to determine the best conditions for extraction of glucocorticoids from hair followed by extract purification on STRATA-X SPE cartridges (Fig. 4). Analytes extraction from ground to fine powder hair shafts instead of cut once yielded ~ 40% and ~ 30–35% increase in R_E_s and ApR_E_s values, respectively. This observation is consistent with other studies^19^ since grounding allows for the break up of the structure of the hair shaft and increases the surface area for extraction. Furthermore, the worst R_E_s for all analytes and slightly better ApR_E_s for cortisol and cortisone were reported when extraction was performed at 56 °C instead of room temperature (Fig. 4). Conducting extraction step at elevated temperature is recommended in some studies^24^. Also, a significant drop in R_E_s and ApR_E_s values was noted when the methanolic extract was directly loaded on the SPE cartridge without evaporation and sample reconstitution in water (aqueous extract). Notably, enhancement of glucocorticoids signals was noted for methanolic extracts (MEs > 100%), while for aqueous extracts signals suppression was observed (MEs < 80%). Based on the obtained results, we decided to pulverize the hair shafts before extraction (performed at room temperature) with methanol and to replace the sample solvent with water before SPE purification.

**Figure 5:**
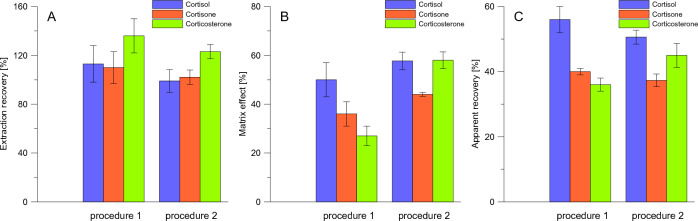
Comparison of extraction recovery (**A**), matrix effect (**B**), and apparent recovery (**C**) obtained during testing of different protocols for elution of glucocorticoids from SPE cartridges and sample concentration before LC–MS/MS analysis. Experiments were conducted using 40 mg of grounded European bison hair sample extracted with 1 mL of methanol (containing 0.05 µg/mL of cortisol, cortisone, and corticosterone) for 24 h at room temperature. Supernatants (800 µL) were evaporated to dryness and redissolved in 1 mL of methanol before being loaded on STRATA-X SPE cartridges. During SPE clean-up, analytes were eluted with 1 mL of methanol/acetonitrile/3 mol/L acetic acid (40:40:20, *v/v/v*) (procedure 1) or methanol (procedure 2). Next, 1 mL of the sample was evaporated to dryness and redissolved in 200 µL of methanol (ultrasonic-assisted step, 2 min)—procedure 1, or its volume was reduced up to 200 µL by evaporation—procedure 2.

Additionally, we provided further improvements on the SPE step: (1) the elution solvent was changed from methanol/acetonitrile/3 mol/L acetic acid (40:40:20, *v/v/v*) to methanol, (2) the eluant was evaporated up to 200 µL instead of complete sample drying. These changes were made to shorten the evaporation step (⁓ 5 times) and avoid possible loss of analytes caused by sample overdrying or insufficient redissolving of the residue. Finally, we received slightly worse R_E_s (but still within acceptable ranges), and less ion suppression caused by hair matrix for steroids, and better ApR_E_s for cortisone and corticosterone (Fig. 5). Furthermore, we reduced sample handling and sample preparation time before LC–MS/MS analysis.

### Method validation

The developed method was validated using hair samples from three different animals (European bison, red squirrel, European hamster) to confirm its flexibility and practical utility. To improve both the accuracy and precision of the obtained results, data for each target compound was normalized on one internal standard—deuterated analog of cortisol (cortisol-D_4_). The implementation of surrogate analytes (e.g. stable-isotope-labeled analogs, SILs) is a popular strategy for reliable quantification of glucocorticoids in different biological matrices including hair^18,24,25^. Undoubtedly, the main disadvantage of this approach is the high cost of SILs. Thus we verified if it is possible to obtain reliable results by applying one SIL for three glucocorticoids, to reduce cost per analysis and develop a protocol attractive for routine analysis.

Depending on the evaluated animal species, the developed method allows for cortisol detection at 0.07–1.19 ng/mL, cortisone at 0.05–0.46 ng/mL, and corticosterone at 0.45–1.26 ng/mL, which corresponds to 3.84 – 29.77 pg/mg, 1.86–11.47 pg/mg, and 11.31–31.51 pg/mg in hair, respectively (Table 2). For all glucocorticoids, the best LODs in hair matrix were noted for the red squirrel and the worst for the European hamster. Furthermore, the method showed good linearity for all analytes in animal hair matrix regardless of species studied and a wide linear range of up to 5 ng/mg. Better LODs were reported in hair collected from the human scalp^24,25,31^ or polar bear^28^. However, the obtained LOQs are sufficient to reliably monitor cortisol, cortisone, and corticosterone in the hair of the study group of free-living animals.

**Table 2:** Comparison of limits of detection (LODs) and limits of quantification (LOQs), and key parameters of matrix-matched calibration curves estimated for glucocorticoids in European bison, Eurasian red squirrel, and European hamster hair matrices.

	Cortisol	Cortisone	Corticosterone
European bison hair			
LOD	0.15 ng/mL (3.84 pg/mg)	0.07 ng/mL (1.84 pg/mg)	0.45 ng/mL (11.31 pg/mg)
LOQ	0.47 ng/mL (11.64 pg/mg)	0.22 ng/mL (5.58 pg/mg)	1.37 ng/mL (34.27 pg/mg)
LR	0.0005–0.2 µg/mL (0.0125–5.0 ng/mg)	0.0003–0.2 µg/mL (0.0075–5.0 ng/mg)	0.0015–0.2 µg/mL (0.0375–5.0 ng/mg)
REq*	*y* = 67.26*x* + 0.051 *y* = 2.69z + 0.051	*y* = 175*x* + 0.013 *y* = 7*z* + 0.013	*y* = 278.1*x* + 0.399 *y* = 11.12*z* + 0.399
R^2^	0.998	0.998	0.997
Eurasian red squirrel hair			
LOD	0.07 ng/mL (1.69 pg/mg)	0.05 ng/mL (1.28 pg/mg)	0.46 ng/mL (11.43 pg/mg)
LOQ	0.21 ng/mL (5.12 pg/mg)	0.16 ng/mL (3.87 pg/mg)	1.39 ng/mL (34.64 pg/mg)
LR	0.0003–0.2 µg/mL (0.0075–5.0 ng/mg)	0.0003–0.2 µg/mL (0.0075–5.0 ng/mg)	0.0015–0.2 µg/mL (0.0375–5.0 ng/mg)
REq*	*y* = 46.48*x* + 0.055 *y* = 1.86*z* + 0.055	*y* = 145.0*x* + 0.055 *y* = 5.80*z* + 0.055	*y* = 299.9*x*-0.664 *y* = 11.99*z*-0.664
R^2^	0.998	0.999	0.996
European hamster hair			
LOD	1.19 ng/mL (29.77 pg/mg)	0.46 ng/mL (11.47 pg/mg)	1.26 ng/mL (31.51 pg/mg)
LOQ	3.61 ng/mL (90.21 pg/mg)	1.39 ng/mL (34.76 pg/mg)	3.82 ng/mL (95.49 pg/mg)
LR	0.0015–0.2 µg/mL (0.0375–5.0 ng/mg)	0.0005–0.2 µg/mL (0.0125 – 5.0 ng/mg)	0.0015–0.2 µg/mL (0.0375–5.0 ng/mg)
REq*	*y* = 79.71*x* + 0.019 *y* = 3.19*z* + 0.019	*y* = 194.90*x* + 0.059 *y* = 7.80*z* + 0.059	*y* = 316.30*x* + 0.947 *y* = 12.65*z* + 0.947
R^2^	0.999	0.999	0.997

*LR* linear range of calibration curve, *REq* regression equation, *R*^2^ coefficient of determination.

*x in µg/mL; *z* in ng/mg.

Regardless of the species evaluated, intraday and interday precision (expressed as RSD) for cortisol, cortisone, and corticosterone remain within the range of 2.84–10.90%, 3.61–12.19%, and 4.24–12.95%, respectively (Table 3). Furthermore, all accuracy data (interday and intraday) were between the acceptable range: 85–115% (Table 3). For European bison hair, the method showed good recoveries within the range of 83.37–104.97% for all glucocorticoids. For other species, in some cases, the exceedance of acceptable levels was observed (R_E_ < 70%). At the sample preparation step, the most valuable loss was noted for corticosterone in red squirrel hair in quality control (QC) samples containing this analyte at low (LQC) and medium (MQC) concentration levels (R_E_s between 41.20 and 47.41%). In QC samples fortified with analytes at high concentration level (HQC), ⁓ 34–40% loss of corticosterone and cortisone were also reported for hair samples from European hamster and red squirrel, respectively (Table 3). Other researchers have reported better R_E_ for corticosterone (~ 80%), and worse for cortisone (~ 68%) and cortisol (~ 55%) in human scalp hairs after pretreatment combining SPE and liquid–liquid extraction (LLE) with toluene^31^. Suppression of steroid hormone signals by hair matrix components has been widely reported^31^. In our study, co-extracted hair matrix components caused suppression of ionization (ME < 100%) for evaluated analytes, and the assessed ME can be classified as medium or strong (Table 3). Only for cortisol determined in European hamster hair samples, MEs were negligible regardless of the studied concentration level. These results implied the complex composition of animal hair and confirmed the need for the additional purification of the methanolic hair extracts before LC–MS/MS analysis. Regarding ME data, the matrix-matched calibration was selected for quantitative purposes. Additionally, the analytes have shown satisfactory stability in final samples (S_FT_ and S_A_ values between 91.40 and 109.73%), which underlines practical usefulness of the developed method (Table 3).

**Table 3:** Validation parameters of the developed method obtained in hair matrix from three different animal species in quality control samples fortified with glucocorticoids at three different concentration levels: low (LQC), medium (MQC), and high (HQC).

	Cortisol			Cortisone			Corticosterone		
	European bison	Eurasian red squirrel	European hamster	European bison	Eurasian red squirrel	European hamster	European bison	Eurasian red squirrel	European hamster
Interday precision [RSD, %]* (n = 3)									
LQC	6.85	6.60	8.74	9.43	10.24	5.17	7.98	12.19	11.20
MQC	7.84	10.90	5.08	8.21	11.46	9.96	5.86	5.66	6.20
HQC	8.10	2.84	9.36	8.37	3.61	6.97	8.84	4.24	7.73
Intraday precision [RSD, %]* (n = 7)									
LQC	9.63	7.11	10.79	12.19	11.14	5.46	7.22	12.95	9.60
MQC	7.75	10.42	9.62	6.75	10.36	8.44	11.70	13.08	9.12
HQC	9.68	4.72	8.91	9.58	5.39	5.65	7.53	5.71	8.70
Interday accuracy [%] (n = 3)									
LQC	97.28	93.18	110.09	108.45	93.18	105.60	109.18	113.23	108.13
MQC	109.86	102.65	93.40	109.57	102.65	92.75	109.91	91.47	108.95
HQC	95.78	103.70	94.78	96.55	96.67	100.70	101.93	101.39	105.92
Intraday accuracy [%]* (n = 7)									
LQC	100.00	95.65	111.10	107.00	94.75	106.79	108.51	106.89	105.90
MQC	109.23	101.55	100.30	108.23	100.06	92.43	108.57	97.84	109.92
HQC	99.74	102.83	98.34	99.66	97.29	99.73	100.17	101.29	107.43
Extraction recovery (R_E_) [%]									
LQC	96.50 ± 1.66	108.90 ± 9.97	70.20 ± 5.17	83.37 ± 7.09	80.29 ± 10.02	86.53 ± 9.88	86.94 ± 4.21	47.41 ± 2.80	91.73 ± 8.22
MQC	104.97 ± 6.42	93.70 ± 4.82	82.40 ± 8.94	99.71 ± 4.66	86.23 ± 5.49	85.68 ± 9.05	104.24 ± 5.47	41.20 ± 1.55	75.40 ± 6.29
HQC	102.53 ± 7.89	91.81 ± 2.65	78.46 ± 3.82	90.89 ± 1.11	66.25 ± 3.63	93.33 ± 8.44	95.74 ± 8.01	104.6 ± 2.05	60.11 ± 3.64
Matrix effect (ME) [%]									
LQC	63.92 ± 7.33	78.03 ± 6.26	98.03 ± 8.73	34.60 ± 1.36	53.51 ± 4.74	51.15 ± 1.76	45.11 ± 4.26	60.52 ± 5.44	68.82 ± 11.16
MQC	54.71 ± 7.85	67.24 ± 4.63	94.08 ± 5.40	47.89 ± 4.85	52.57 ± 1.73	58.31 ± 2.00	51.48 ± 3.74	56.44 ± 9.89	64.65 ± 4.83
HQC	48.63 ± 8.46	54.82 ± 6.39	94.66 ± 4.84	42.80 ± 4.21	63.62 ± 5.84	52.20 ± 5.18	52.77 ± 6.80	60.40 ± 5.58	60.11 ± 3.64
Autosampler stability (S_A_) [%]									
LQC	95.34 ± 2.68	103.64 ± 5.49	101.96 ± 6.62	97.81 ± 9.20	106.20 ± 6.70	96.65 ± 4.74	104.37 ± 3.52	100.70 ± 3.29	103.62 ± 0.33
MQC	92.86 ± 6.56	96.09 ± 1.74	104.36 ± 11.43	98.16 ± 4.32	95.35 ± 3.07	94.24 ± 1.81	104.12 ± 6.49	94.58 ± 4.09	107.70 ± 5.42
HQC	101.12 ± 3.22	109.40 ± 1.20	109.73 ± 2.09	99.60 ± 5.08	108.89 ± 4.19	100.39 ± 12.50	101.49 ± 2.39	110.81 ± 3.06	104.27 ± 3.02
Freeze–thaw stability (S_FT_) [%]									
LQC	96.11 ± 2.74	98.19 ± 9.23	104.46 ± 8.40	102.55 ± 8.04	97.66 ± 7.70	96.21 ± 1.42	109.41 ± 3.93	91.40 ± 3.98	102.03 ± 0.28
MQC	96.52 ± 6.38	93.64 ± 6.09	94.51 ± 1.37	103.51 ± 3.69	92.27 ± 8.73	103.79 ± 7.65	104.15 ± 2.35	97.54 ± 9.45	92.72 ± 6.68
HQC	94.50 ± 2.67	101.12 ± 1.50	92.99 ± 3.67	98.52 ± 7.45	101.26 ± 4.43	109.37 ± 2.14	98.05 ± 1.02	102.66 ± 2.10	93.06 ± 2.45

Concentrations of analytes in QC samples: Cortisol: LQC = 0.038 ng/mg (European bison and red squirrel hair) or 0.125 ng/mg (European hamster hair); MQC = 0.25 ng/mg; HQC = 4.0 ng/mg; Costisone: LQC = 0.038 ng/mg; MQC = 0.25 ng/mg; HQC = 4.0 ng/mg; Corticosterone: LQC = 0.125 ng/mg; MQC = 0.25 ng/mg; HQC = 4.0 ng/mg.

### Analysis of animal hair samples

Quantitative results of the concentration of cortisol, cortisone, and corticosterone in hair samples collected from European bison, European hamster, and Eurasian red squirrel are summarized in Table 4 and supported by the examples of LC–MS/MS data in Supplementary Fig. 1S.

**Table 4:** The concentration of cortisol, cortisone, and corticosterone obtained during LC–MS/MS analysis of experimental hair samples from free-living European bison, European hamster, and Eurasian red squirrel.

Species	Individual sample number	Hair cortisol concentration [pg/mg ± SD]	Hair cortisone concentration [pg/mg ± SD]	Hair corticosterone concentration [pg/mg ± SD]
European bison	S1	ND	15.13 ± 1.63	ND
	S2	12.40 ± 0.17	ND	ND
	S3	ND	7.96 ± 1.70	ND
	S4	ND	5.66 ± 0.08	ND
	S5	16.80 ± 2.59	5.90 ± 0.53	ND
	S6	ND	6.66 ± 0.82	ND
	S7	29.49 ± 2.29	D	ND
	S8	ND	ND	ND
	S9	18.12 ± 1.53	D	ND
	S10	10.93 ± 1.22	7.55 ± 0.12	ND
European hamster	S1	ND	ND	ND
	S2	ND	ND	139.38 ± 3.48
	S3	ND	ND	142.30 ± 6.05
Eurasian red squirrel	S1	69.35 ± 2.11	34.07 ± 2.38	ND
	S2	49.65 ± 1.55	42.63 ± 2.36	ND
	S3	109.30 ± 5.54	61.90 ± 3.85	ND
	S4	83.84 ± 6.05	75.42 ± 2.30	ND
	S5	50.28 ± 5.01	68.20 ± 6.85	ND
	S6	19.82 ± 1.67	69.65 ± 4.53	ND
	S7	25.92 ± 2.51	61.30 ± 1.21	ND
	S8	10.93 ± 1.14	49.80 ± 3.20	ND
	S9	215.15 ± 10.04	91.91 ± 2.20	ND
	S10	118.36 ± 3.22	96.31 ± 1.08	ND

*ND* “not detected”; *D* detected, not quantified.

**Figure 6:**
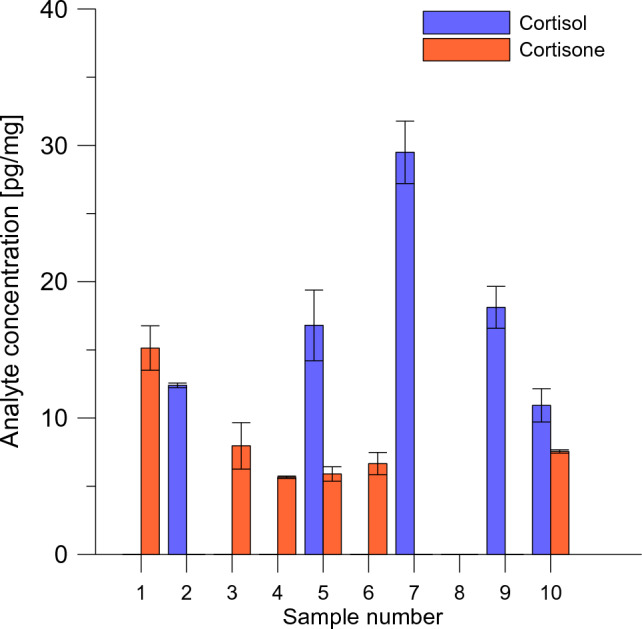
Concentration of cortisol and cortisone in hair samples from 10 European bison individuals. Corticosteroids were not consistently present in all samples, and the concentration of cortisol did not correlate with cortisone.

The positive values obtained for the tested glucocorticoids ranged from 5.7 to 215.2 pg/mg, however, ND (i.e., “not detected”) cases were also obtained. In most cases, a “not detected” result is expected and is not related to the quality of the method. It is known that in certain animal species, the metabolic pathway of cortisol (in European bison and red squirrel) or corticosterone (in European hamster) is dominant. In this context, the lack of e.g., corticosterone in the hair of European bison is a correct and expected result. However, “not detected” results for the cortisol-cortisone pair of hormones in animals where cortisol is the primary glucocorticoid should be interpreted differently. It is known that cortisol can be reversibly transformed to its inactive metabolite—cortisone and the detection of both steroids is likely. For example, in the red squirrel, both hormones were found in significant concentrations in every sample tested. In the European bison hair, however, either one or the other glucocorticoid (more often cortisone) was detected (Table 4; Fig. 6). If only one of the hormones (cortisol or cortisone) were utilized for the research, entirely different animals would be identified as potentially the most susceptible to the analyzed stressors. The obtained results for European bison show how important it is for the correct assessment of stress reactions of protected animal species to simultaneously determine several glucocorticoids from a given metabolic pathway. Our method clearly shows that measuring only cortisol could lead to erroneous conclusions about which individual has higher levels of stress hormones. Such a result may be an important clue in studies that use only enzyme immunoassays and determine only a single compound. As our method shows, at least for some species the set of analyzed makers should be extended.

## Materials and methods

### Materials and reagents

Ultrapure water was produced by a Milli-Q water purification system (Merck Millipore, Darmstadt, Germany). Hypergrade isopropanol, acetonitrile, formic acid, and acetic acid were from Merck (Darmstadt, Germany), and methanol was from POCH (Gliwice, Poland). Standards of cortisol, cortisone, corticosterone, and cortisol-D_4_ were from Sigma-Aldrich (Saint Louis, Missouri, USA). Stock and working solutions of standards were prepared in methanol and stored at − 20 °C before use.

For SPE purification of hair extracts, the Strata-X cartridges (Polymeric Reversed Phase, particle size: 33 µm; 30 mg/1 mL; pore size: 85 Å; pore volume: 1.2 mL/g; surface area: 800 m^2^/g, p.n. 8B-S100-TAK) supplied by Phenomenex (Aschaffenburg, Germany) were used. During the method development, we also tested SPE cartridges provided by Supelco (Bellefonte, PA, USA): (1) Discovery SPE Reversed-Phase DSC-8 (100 mg/1 mL, p.n. 52707-U, monomerically bonded, octyl (9%C)), (2) Discovery SPE Reversed-Phase DSC-18 (100 mg/1 mL, p.n. 52602-U, polymerically bonded, octadecyl (18%C)), (3) Discovery SPE Reversed-Phase DSC-18Lt. (100 mg/1 mL, p.n. 52611-U, monomerically bonded, octadecyl (11%C)), (4) Discovery DSC-Ph (100 mg/1 mL, p.n. 52725-U, monomerically bonded, phenyl (7%C)), (5) Discovery DSC-CN (100 mg/1 mL, p.n. 52694-U, monomerically bonded, cyanopropyl (7%C)), (6) Discovery DSC-MCAX (100 mg/1 mL, p.n. 2616-U, octyl and benzene sulfonic acid bondings), (7) Supel ™-Select HLB SPE (30 mg/1 mL, p.n. 54181-U) and Thermo Fisher Scientific (Waltham, USA), (8) SOLA HRP (10 mg/1 mL, p.n. 60109-001).

The universal kits of dSPE (Agilent Bond Elut p.n. 5982-0028, 5982-5221, 5982-5321, 5982-5022) were purchased from Agilent Technologies (Folsom, USA). The composition of dSPE kits was as follows: dSPE 1: 150 mg MgSO_4_ + 50 mg PSA; dSPE 2: 150 mg MgSO_4_ + 25 mg PSA + 7.5 mg GCB; dSPE 3: 150 mg MgSO_4_ + 25 mg PSA + 2.5 mg GCB; dSPE 4: 150 mg MgSO_4_ + 50 mg PSA + 7.5 mg GCB + 50 mg C18.

### Hair samples characteristics

The hair samples used for this study came from a wild animal tissue bank existing at the following universities in Poland (central Europe): Warsaw University of Life Sciences (consent of Regional Directorate for Nature Protection in Warsaw for red squirrel number WPN-I.6401.208.2018.PF, and for European bison number WPN-I.6401.90.2014.EB.1) and the John Paul II Catholic University of Lublin (consent of Regional Directorate for Nature Protection in Lublin number: WPN.6401.3.1.2023.AO). No animal was intentionally captured or killed for this work. For each sample, information was available on the sex, body weight, location of the animal, and the time when the samples were collected (Table 5). The hair samples were stored in string plastic bags in the − 24 °C freezer. The samples were obtained from animals by cutting the hair close to the skin, and these samples lacked hair follicles.

**Table 5:** Characteristics of the samples used for analysis include information on the animal species from which the hair samples were collected, the animal's sex, age or body weight, its approximate location and the date of sampling.

Species	Individual sample number	Sex	Age or body mass	The location of the sample's origin	Date of sample collection
European bison	S1	F	0.5-year-old	Bieszczady	September, 2021
	S2	M	18-year-old		May, 2021
	S3	M	10-year-old		
	S4	F	12-year-old		March, 2022
	S5	F	2-year-old	Kiermusy	June, 2021
	S6	M	2-year-old	Niepołomice	
	S7	M	2-year-old		
	S8	F	6-year-old	Bieszczady	May, 2021
	S9	M	0.5-year-old	Białowieża	January, 2022
	S10	M	17-year-old	Bieszczady	August, 2021
European hamster	S1	F	206 g	The surroundings of Lublin	August, 2019
	S2	M	150 g		
	S3	M	158 g		July, 2020
Eurasian red squirrel	S1	M	250 g	A city park in the center of Warsaw	May, 2019
	S2	M	310 g		
	S3	F	370 g		
	S4	M	370 g	An urban forest situated on the outskirts of Warsaw	July, 2019
	S5	F	370 g		
	S6	M	270 g		September, 2019
	S7	M	340 g		
	S8	F	360 g		
	S9	M	340 g		
	S10	F	270 g	A city park in the center of Warsaw	

### Optimization of LC–MS/MS parameters

In initial conditions, the scan spectra of target analytes (including internal standard) were acquired in ESI + to select the precursor ions. ESI in positive polarity allows for the detection of low levels of glucocorticoids using LC–MS/MS in different biological matrices^18,21,24^. The ions corresponding to [M + H]^+^ were selected to develop the quantitative method. Next, the fragmentor voltage (60–160 V) and collision energy (0–40 eV) were optimized to provide appropriate conditions for the formation of precursor ions. The obtained data were inspected to select characteristic transitions and selectively detect analytes in a multiple reaction monitoring (MRM) mode. Next, key parameters of the mass spectrometer (MS) were optimized to achieve good sensitivity of measurement: nebulizer pressure, drying gas temperature, drying gas flow, sheath gas temperature, sheath gas flow, capillary voltage, and nozzle voltage within the range of 30–45 psi, 250–350 °C, 5–9 L/min, 250–400 °C, 5–10 L/min, 3000–5000 V, and 0–900 V, respectively.

### Hair sample preparation

Hair strands were washed twice by hand shaking in 5 mL of isopropanol for 3 min to remove the extrinsic contamination from their surface. The washing solutions were decanted and removed, and hair samples were dried overnight at room temperature. After this step, the hair was carefully cut with surgical scissors into ⁓ 5 mm pieces. Then, ~ 40 mg of hair sample was accurately weighed using the OHAUS Adventrer (Parsippany, New Jersey, USA), transferred into a 2 mL plastic tube, and milled at 3000 rpm with five steel beads (3 mm) for 9 min (in three cycles of 3 min each). The powdered hair sample was incubated in 1 mL of methanol containing 10 µg/mL of cortisol-D_4_ at room temperature using a Stuart SSL4 see-saw rocker (Vernon Hills, Illinois, USA) at 70 rpm speed range. After 24 h, the samples were centrifuged (13 000 rpm, 15 min) using a 5415R centrifuge (Eppendorf, Germany), and supernatants (800 µL) were transferred to a new centrifugal tube. Then, aliquots were evaporated to dryness using an EZ-2 Elite Personal Evaporator (Genevac Ltd., Ipswich, UK), reconstituted in 1 mL of ultrapure water, and sonicated for 5 min in an ultrasonic bath (Polsonic, Warsaw, Poland). Next, the obtained samples were purified by SPE using a Gilson GX-271 ASPEC (Middleton, WI, USA) automatic extraction system operated by Gilson Ethernet Utility 1.8.6.1 and Trilution LH 2.0 software. The Strata-X SPE cartridges were conditioned with 1 mL of methanol, and equilibrated with 1 mL of water, respectively. Then 1 mL of the sample was loaded onto the cartridge, which was further washed with 1 mL of water. Elution was carried out with 1 mL of methanol. The eluate was dried up to 200 µL. The clear supernatant was transferred into a glass chromatographic insert vial and analyzed in triplicate by the LC–MS/MS method. The injection volume was 10 µL.

### LC–MS/MS analysis of hair extracts

Measurements were carried out on a 1290 infinity UHPLC system consisting of a degasser, binary pump, autosampler, and column thermostat (Agilent Technologies, Santa-Clara, CA, USA). The UHPLC instrument set-up was connected with an Agilent 6460 triple quadrupole mass spectrometer (QQQ) equipped with an Agilent Jet Stream electrospray ion (ESI) source. Data were acquired and analyzed with Agilent MassHunter Acquisition software v.B.08 and Agilent MassHunter Quantitative Analysis software v.B.07, respectively. Chromatographic separation was achieved at 40 °C on the Zorbax Eclipse Plus C18 RRHT column (2.1 mm × 100 mm × 1.8 µm) protected by the Zorbax Eclipse Plus C18 guard column (2.1 mm × 12.5 mm × 1.8 µm) both from Agilent Technologies. Gradient elution was performed using a mobile phase consisting of 0.1% (*v/v*) formic acid in water (solvent A) and methanol (solvent B) at the flow rate of 0.35 mL/min and the following program: 0–4 min 20–60% solvent B; 4–5 min 60% solvent B; 5–7 min 60–70% solvent B (post run: 2 min). The MS conditions were optimized using Source and iFunnel Optimizer software v.B.08.00 (Agilent Technologies). Data were acquired in dynamic multiple reaction monitoring (dMRM) mode in positive polarity using settings detailed in Table 1.

An amount of individual glucocorticoid in authentic animal hair samples was determined from the corresponding matrix-matched calibration curve. Next, the values in µg/mL were converted to µg/g considering the mass of hair strands subjected to milling and extraction. The data were expressed as the mean value from three injections of the sample plus the standard deviation.

### Method validation

For validation experiments, representative hair pools originating from different individuals showing undetectable (from European hamster and European bison) or low basal levels (for red squirrel) of target glucocorticoids. A series of quality control (QC) samples were prepared to evaluate: linearity, the limit of detection (LOD), the limit of quantification (LOQ), accuracy, precision, extraction recovery (R_E_), autosampler stability (S_A_), the freeze–thaw stability (S_FT_), and matrix effect (ME). Blank (unspiked samples) were also analyzed, and the peak area corresponding to the endogenous concentration of the analyte in the hair pool was subtracted from the peak area detected in the QC sample. Acceptance criteria were taken from guidance published by FDA^32^.

For linearity evaluation, nine matrix-matched calibration standards with increasing concentrations of glucocorticoids in the range of 0.3 ng/mL to 0.2 µg/mL were analyzed. Each sample was fortified with a constant amount of internal standard. Next, the regression plots were built for each analyte using the response factors—the ratio of the analyte peak area over cortisol-D_4_ peak area. Linearity was acceptable with the coefficient of determination (R^2^) of more than 0.99. For each animal species, LODs and LOQs were calculated independently using the formula: LOD or LOQ = a × SD/b, where *a* is a factor of 3.3 or 10 for LOD and LOQ, respectively, *SD* is a standard deviation of the intercept and *b* is a slope of the regression line.

Accuracy, precision, stability, R_E_ and ME were studied for hair matrix using QC samples fortified with analytes at three different concentration levels: low (LQC =  ~ 3 × LOQ), medium (MQC), and high (HQC). Interday and intraday variations of accuracy (trueness) and precision (repeatability) were calculated using data obtained from 3 or 7 individually prepared QC samples analyzed within one day or on three different days, respectively. The precision was expressed as the relative standard deviation (RSD), while accuracy was calculated using ratios of analyte concentrations determined in the samples from the corresponding calibration curve (A) to concentration added (B) (accuracy = A/B × 100%). Acceptable levels of accuracy and precision were within 15%. For R_E_, the ratios of peak areas in pre-extraction (C) and post-extraction (D) spiked pooled hair matrix were compared (R_E_ = (C/D) × 100%; acceptable range: 70–120%). For ME calculation, the ratios of peak areas in post-extraction spiked hair sample (D) to corresponding spiked neat solvent (E) were compared (ME = (D/E) × 100%). ME values within 0–10%, 10–20%, 20–50%, and < 50% were classified as negligible, soft, medium, and strong, respectively. The stability was estimated by comparing signals of glucocorticoids in freshly prepared hair extracts to samples stored for one day at 25 °C in the autosampler tray (for S_A_) or samples subjected to three freeze/thaw cycles from 25 to − 20 °C (for S_FT_). Acceptable levels of stability were within ± 10%. Each QC sample was injected three times.

### Analysis of hair samples from free-living animals

To demonstrate the applicability of the developed analytical method, the determinations of glucocorticoids in hair samples from free-living animals of selected species were carried out. We chose three mammal species with a high conservation status for our study. Protected species and their welfare are frequent subjects of research, making the development of dedicated analytical methods appealing to a broader audience. We selected animals from various taxonomic groups: European bison, European hamster, and Eurasian red squirrel. These animals differed in terms of body size, lifestyle (including terrestrial, burrowing, and arboreal species), social organization (either living in herds or solitary), and the expected type of hair glucocorticoids.

European bison is a symbolic species for nature conservation in Europe. It is the largest living mammal in Europe. This species became extinct in the wild at the beginning of the twentieth century, but thanks to many conservation efforts, its population has been restored and currently there are about 10,000 European bison in Europe, including nearly 80% in the wild. This species occurs mainly in forests, but it also willingly uses open areas, which provide it with an attractive food base^33–35^. Due to various threats^36–38^, European bison populations are constantly monitored and the measurement of glucocorticoids can be a good tool that can be routinely used in monitoring. The development of an analytical method for measuring corticoids in hair for this species is therefore very justified from a practical point of view.

European hamster is a burrowing rodent species with high conservation status in the European Union. It is strictly protected in many European countries, listed in EU legislation (Habitats Directive) and international conventions (Appendix II of the Bern Convention). This species is totally dependent on agriculture because farmlands and human settlements occupy its original fertile steppe habitats^39^. Nowadays, many research and conservation actions are being taken for this mammal^40–45^. For this reason, the measurement of glucocorticoids can be a useful tool that can be routinely used in the monitoring of endangered or reintroduced hamster populations.

The Eurasian red squirrel is also a protected species in Poland. Due to the occupation of urbanized areas, this species is exposed to various anthropogenic stressors^46^. At the same time, in some European countries, this species is under pressure from the geographically alien (originating from North America) eastern grey squirrel *Sciurus carolinensis*, which is also a carrier of the highly pathogenic to red squirrels parapoxvirus^47^. It is also known from the literature that squirrels are characterized by quite high levels of steroids^48–50^ and thus can be a very useful species to test analytical methods as a reference point for other species.

## Conclusions

An accurate LC–MS/MS method for the determination of cortisol, cortisone, and corticosterone in animal hair was developed and validated. The method flexibility was evaluated on hair samples of three different species of mammals with high conservation status and different ecological features. The method comprises a simple extraction with methanol and further sample clean-up using solid-phase extraction (SPE). A special emphasis was put on the purification of hair extracts to reduce the suppression of the glucocorticoid’s signals caused by the matrix background. Numerous commercially available SPE cartridges and dispersive SPE kits were selected for hair extract purification. The method performance was evaluated on representative hair pools delivered from selected mammal species by determining key validation parameters.

With increasing human pressure and climate change, there is an urgent need to develop validated tools for reliable assessment and monitoring of the environmental stressors for various free-living animals. This allows for a better understanding of contemporary natural processes and taking effective remedial action. The method presented in this work demonstrates that keratinized tissues of animals can be successfully utilized for research, and measuring multiple glucocorticoids simultaneously provides more comprehensive information than solely concentrating on one of them. However, the utility of specific glucocorticoids as markers of the stress response in keratinized tissues should be substantiated by additional experimental studies on specific animal targets. During these studies, possible pathways of glucocorticoid deposition in hair, as well as species differences in the hair growth and shedding cycle, should be considered. Attention should also be given to the potential specificity of glucocorticoid deposition in the hair of hibernating species, where samples collected in spring may reflect the free corticosterone concentration from the hair growth period before hibernation.

### Supplementary Information

Supplementary Figure S1.

## Data Availability

All data generated or analysed during this study are included in this published article.

## References

[1] Botía M (2023). Different types of glucocorticoids to evaluate stress and welfare in animals and humans: General concepts and examples of combined use. Metabolites.

[2] Burnard C, Ralph C, Hynd P, Edwards JH, Tilbrook A (2016). Hair cortisol and its potential value as a physiological measure of stress response in human and non-human animals. Anim. Prod. Sci..

[3] Ralph CR, Tilbrook AJ (2016). Invited review: The usefulness of measuring glucocorticoids for assessing animal welfare. J. Anim. Sci..

[4] Sheriff MJ, Dantzer B, Delehanty B, Palme R, Boonstra R (2011). Measuring stress in wildlife: Techniques for quantifying glucocorticoids. Oecologia.

[5] Contreras ET, Vanderstichel R, Hovenga C, Lappin MR (2021). Evaluation of hair and nail cortisol concentrations and associations with behavioral, physical, and environmental indicators of chronic stress in cats. J. Vet. Intern. Med..

[6] George SC, Smith TE, Mac Cana PSS, Coleman R, Montgomery WI (2014). Physiological stress in the Eurasian badger (*Meles meles*): Effects of host, disease and environment. Gen. Comp. Endocrinol..

[7] Shi R (2021). Genetic parameters of hair cortisol as an indicator of chronic stress under different environments in Holstein cows. J. Dairy Sci..

[8] Gao W, Kirschbaum C, Grass J, Stalder T (2016). LC–MS based analysis of endogenous steroid hormones in human hair. J. Steroid Biochem. Mol. Biol..

[9] Wester VL, van Rossum EFC (2015). Clinical applications of cortisol measurements in hair. Eur. J. Endocrinol..

[10] Fokidis HB (2016). Sources of variation in plasma corticosterone and dehydroepiandrosterone in the male northern cardinal (Cardinalis cardinalis): I. Seasonal patterns and effects of stress and adrenocorticotropic hormone. Gen. Comp. Endocrinol..

[11] Baxter-Gilbert JH, Riley JL, Mastromonaco GF, Litzgus JD, Lesbarrères D (2014). A novel technique to measure chronic levels of corticosterone in turtles living around a major roadway. Conserv. Physiol..

[12] Macbeth BJ, Cattet MRL, Stenhouse GB, Gibeau ML, Janz DM (2010). Hair cortisol concentration as a noninvasive measure of long-term stress in free-ranging grizzly bears (*Ursus arctos*): Considerations with implications for other wildlife. Can. J. Zool..

[13] Crain DD, Karpovich SA, Quakenbush L, Polasek L (2021). Using claws to compare reproduction, stress and diet of female bearded and ringed seals in the Bering and Chukchi seas Alaska between 1953–1968 and 1998–2014. Conserv. Physiol..

[14] Fokidis HB, Brock T, Newman C, Macdonald DW, Buesching CD (2023). Assessing chronic stress in wild mammals using claw-derived cortisol: A validation using European badgers (*Meles meles*). Conserv. Physiol..

[15] Colding-Jørgensen P, Hestehave S, Abelson KSP, Kalliokoski O (2020). Hair glucocorticoids are not a historical marker of stress—exploring the time-scale of corticosterone incorporation into hairs in a rat model. bioRxiv.

[16] Klich D (2021). Stress hormone level and the welfare of captive European bison (*Bison bonasus*): The effects of visitor pressure and the social structure of herds. Acta Vet. Scand..

[17] Matas D, Keren-Rotem T, Koren L (2016). A method to determine integrated steroid levels in wildlife claws. Gen. Comp. Endocrinol..

[18] Šimková M, Kolátorová L, Drašar P, Vítků J (2022). An LC-MS/MS method for the simultaneous quantification of 32 steroids in human plasma. J. Chromatogr. B.

[19] Meyer J, Novak M, Hamel A, Rosenberg K (2014). Extraction and analysis of cortisol from human and monkey hair. J. Vis. Exp..

[20] Dasenaki M (2019). Simultaneous determination of free cortisol, cortisone and their tetrahydrometabolites in urine by single solvent extraction and liquid chromatography–tandem mass spectrometry. Anal. Lett..

[21] Saluti G (2022). Determination of hair cortisol in horses: Comparison of immunoassay vs LC-HRMS/MS. Anal. Bioanal. Chem..

[22] Palme R, Rettenbacher S, Touma C, El-Bahr SM, Möstl E (2005). Stress hormones in mammals and birds: Comparative aspects regarding metabolism, excretion, and noninvasive measurement in fecal samples. Ann. New York Acad. Sci..

[23] Jewgenow K, Azevedo A, Albrecht M, Kirschbaum C, Dehnhard M (2020). Hair cortisol analyses in different mammal species: Choosing the wrong assay may lead to erroneous results. Conserv. Physiol..

[24] Voegel CD, Baumgartner MR, Kraemer T, Wüst S, Binz TM (2021). Simultaneous quantification of steroid hormones and endocannabinoids (ECs) in human hair using an automated supported liquid extraction (SLE) and LC-MS/MS—Insights into EC baseline values and correlation to steroid concentrations. Talanta.

[25] Dong Z, Wang C, Zhang J, Wang Z (2017). A UHPLC-MS/MS method for profiling multifunctional steroids in human hair. Anal. Bioanal. Chem..

[26] Binz TM (2018). Systematic investigations of endogenous cortisol and cortisone in nails by LC-MS/MS and correlation to hair. Anal. Bioanal. Chem..

[27] Millspaugh JJ, Washburn BE (2004). Use of fecal glucocorticoid metabolite measures in conservation biology research: Considerations for application and interpretation. Gen. Comp. Endocrinol..

[28] Weisser JJ (2016). A novel method for analysing key corticosteroids in polar bear (*Ursus maritimus*) hair using liquid chromatography tandem mass spectrometry. J. Chromatogr..

[29] Francesco JD (2021). Qiviut cortisol reflects hypothalamic–pituitary–adrenal axis activity in muskoxen (*Ovibos moschatus*). Gen. Comp. Endocrinol..

[30] Caslini C (2016). Use of hair cortisol analysis for comparing population status in wild red deer (*Cervus elaphus*) living in areas with different characteristics. Eur. J. Wildl. Res..

[31] Koskivuori J (2022). A quantitative ultra-performance liquid chromatography high-resolution mass spectrometry analysis of steroids from human scalp hair. J. Pharm. Biomed. Anal..

[32] Food and Drug Administration. *Bioanalytical Method Validation. Guidance for Industry* (2018).

[33] Krasińska, M., Krasiński, Z. A., Olech, W. & Perzanowski, K. European bison. In *Ecology, Evolution and Behaviour of Wild Cattle: Implications for Conservation* (ed. Melletti, M.) 115–173 (Cambridge University Press, 2014).

[34] Perzanowski K, Januszczak M, Łopucki R (2019). Historical changes in land use influence current habitat preferences of large herbivores. Landsc. Ecol..

[35] Łopucki R (2023). Individual differentiation of habitat preferences indicate high flexibility in habitat use by European bison (*Bison bonasus*). Glob. Ecol. Conserv..

[36] Klich D (2020). Pesticides and conservation of large ungulates: Health risk to European bison from plant protection products as a result of crop depredation. PLoS One.

[37] Klich D, Kitowski I, Łopucki R, Wiącek D, Olech W (2021). Essential differences in the mineral status of free-ranging European bison *Bison bonasus* populations in Poland: The effect of the anthroposphere and lithosphere. Sci. Total Environ..

[38] Larska, M. & Krzysiak, M. K. Infectious disease monitoring of European Bison (*Bison bonasus*). In *Wildlife Population Monitoring* 248–269 (2019). 10.5772/intechopen.84290.

[39] Ziomek J, Banaszek A (2007). The common hamster, *Cricetus cricetus* in Poland: status and current range. Folia Zool..

[40] Łopucki R, Perzanowski K (2018). Effects of wind turbines on spatial distribution of the European hamster. Ecol. Indic..

[41] Flamand A (2019). Hamsters in the city: A study on the behaviour of a population of common hamsters (*Cricetus cricetus*) in urban environment. PLoS One.

[42] Łopucki R, Kitowski I (2019). Local democracy does not support conservation of an urban population of the European hamster *Cricetus cricetus*. Oryx.

[43] Surov AV (2019). Circle of life: the common hamster (*Cricetus cricetus*) adaptations to the urban environment. Integr. Zool..

[44] La Haye MJJ, van Kats RJM, Müskens GJDM, Hallmann CA, Jongejans E (2020). Predation and survival in reintroduced populations of the Common hamster *Cricetus cricetus* in the Netherlands. Mamm. Biol..

[45] Feoktistova NY, Meschersky IG, Karmanova TN, Gureeva AV, Surov AV (2022). Allele diversity of the major histocompatibility complex in the common hamster (*Cricetus cricetus*) in urban and rural populations. Biol. Bull..

[46] Krauze-Gryz D, Gryz J, Brach M (2021). Spatial organization, behaviour and feeding habits of red squirrels: Differences between an urban park and an urban forest. J. Zool..

[47] Rushton SP (2006). Disease threats posed by alien species: The role of a poxvirus in the decline of the native red squirrel in Britain. Epidemiol. Infect..

[48] Cordeschi G, Peric T, Prandi A, Zoratto F, Mori E (2021). Environmental variability and allostatic load in the Eurasian red squirrel *Sciurus vulgaris*. Rend. Lincei. Sci. Fis. e Nat..

[49] Haigh A, Butler F, O’Riordan R, Palme R (2017). Managed parks as a refuge for the threatened red squirrel (*Sciurus vulgaris*) in light of human disturbance. Biol. Conserv..

[50] Dantzer B (2016). Measurement of fecal glucocorticoid metabolite levels in Eurasian red squirrels (*Sciurus vulgaris*): Effects of captivity, sex, reproductive condition, and season. J. Mammal..

