# Supplementary material for: A validated LC–MS/MS method for simultaneous determination of key glucocorticoids in animal hair for applications in conservation biology
Source: Sci Rep. 2023 Dec 28;13:23089. doi: 10.1038/s41598-023-49940-2 (PMC10754919; doi:10.1038/s41598-023-49940-2)
Supplement: Supplementary file 1 — Supplementary Figure S1. [file 41598_2023_49940_MOESM1_ESM.docx]

**A validated LC-MS/MS method for simultaneous determination of key glucocorticoids in animal hair for applications in conservation biology**

Ilona Sadok^1,^*, Kinga Ożga^2^, Daniel Klich^3^, Wanda Olech^3^, Dagny Krauze-Gryz^4^, Agata Beliniak^4^, Rafał Łopucki^2^

^1^Department of Chemistry, Institute of Biological Sciences, Faculty of Medicine, The John Paul II Catholic University of Lublin, Konstantynów 1J, 20-708 Lublin, Poland

^2^Department of Biomedicine and Environmental Research, Institute of Biological Sciences, The John Paul II Catholic University of Lublin, Konstantynów 1J, 20-708 Lublin, Poland

^3^Department of Animal Genetics and Conservation, Warsaw University of Life Sciences, Ciszewskiego 8,02-786 Warsaw, Poland

^4^Department of Forest Zoology and Wildlife Management, Warsaw University of Life Sciences, Nowoursynowska 159, 02-776 Warsaw, Poland

*Corresponding author: ilona.sadok@kul.pl (I. Sadok), **phone:** +48 81 445 46 18

Ilona Sadok: ilona.sadok@kul.pl; ORCID: 0000-0003-1154-7581

Kinga Ożga: kingaozga@kul.pl

Daniel Klich: daniel_klich@sggw.edu.pl, ORCID: 0000-0001-6276-2316

Wanda Olech: wanda_olech@sggw.edu.pl, ORCID: 0000-0002-6166-3954

Dagny Krauze-Gryz: dagny_krauze_gryz@sggw.edu.pl

Agata Beliniak: agata_beliniak@sggw.edu.pl

Rafał Łopucki:rafal.lopucki@kul.pl, ORCID: 0000-0003-2137-8742

|  | Cortisone | Cortisol-D4 | Cortisol | Corticosterone |
| --- | --- | --- | --- | --- |
| A |  |  |  |  |
| B |  |  |  |  |
| C |  |  |  |  |

**Fig. 1S.** Example of MRM data obtained during LC-MS/MS analysis of hair samples from (A) European bison, (B) Eurasian red squirrel, and (C) European hamster.
